# American Basil, *Ocimum americanum*, Has Neuroprotective Properties in the Aging Process

**DOI:** 10.3390/nu17142368

**Published:** 2025-07-19

**Authors:** Ionara Rodrigues Siqueira, Cláudia Vanzella, Gisele Agustini Lovatel, Karine Bertoldi, Christiano Spindler, Felipe dos Santos Moysés, Adriana Vizuete, Gilsane Lino von Poser, Carlos Alexandre Netto

**Affiliations:** 1 Programa de Pós-Graduação em Ciências Biológicas: Fisiologia, Instituto de Ciências Básicas da Saúde, Universidade Federal do Rio Grande do Sul, Porto Alegre 90035-003, RS, Brazil; kakibertoldi@gmail.com (K.B.); christianospindler@gmail.com (C.S.); felipemoyses@gmail.com (F.d.S.M.); 2 Programa de Pós-Graduação em Ciências Biológicas: Bioquímica, Instituto de Ciências Básicas da Saúde, Universidade Federal do Rio Grande do Sul, Porto Alegre 90035-003, RS, Brazil; cvanzella@gmail.com (C.V.); adriana.vizuete@ufcspa.edu.br (A.V.); alexneto@ufrgs.br (C.A.N.); 3 Programa de Pós-Graduação em Ciências Biológicas: Neurociências, Instituto de Ciências Básicas da Saúde, Universidade Federal do Rio Grande do Sul, Porto Alegre 90035-003, RS, Brazil; gilovatel@gmail.com; 4 Departamento de Produção de Matéria Prima, Universidade Federal do Rio Grande do Sul, Porto Alegre 90610-000, RS, Brazil; gilsane.von@ufrgs.br

**Keywords:** americanum basil, functional food, hippocampus, neuroprotection, antioxidant activity, anti-inflammatory action

## Abstract

**Background/Objectives**: There is evidence concerning herbal medicines and plant-based compounds, including Lamiaceae species, as putative senolytic agents; however, there are only a few reports on *Ocimum americanum* properties using rat models. The aim of this study was to investigate the neuroprotective effects and potential modes of action of *Ocimum americanum* L. using ex vivo and in vivo assays to assess the effects of OAEE on hippocampal tissue from young adult and late middle-aged Wistar rats, with a focus on oxidative stress, cholinesterase activity, and neuroinflammatory markers. **Methods**: *Ocimum americanum* ethanol extract (OAEE) was incubated with hippocampal slices of young adult and late middle-aged male Wistar rats exposed to H_2_O_2_; an acute treatment with OAEE was evaluated in aversive memory performance and neurochemical parameters, such as hippocampal cellular oxidative state, and anticholinesterase activity, and a diet supplementation of OAEE were evaluated on several hippocampal biochemical parameters, such as oxidative state, anticholinesterase activity, and neuroinflammatory parameters in young adult and late middle-aged male rats. **Results**: OAEE reversed the H_2_O_2_-induced impaired cellular viability in hippocampal slices from young adult rats, as well as protected hippocampal slices against H_2_O_2_-induced damage in both young adult and late middle-aged Wistar rats, indicating its neuroprotective action. Chronic dietary OAEE supplementation reduced aging-induced increases in reactive species and lipid peroxidation levels in the hippocampus. Indeed, this supplementation reduced the TNF-α content in hippocampus from both ages, and IL-1β levels in young adult rats. **Conclusions**: The antioxidant actions of OAEE here observed, preventing the lipoperoxidation, as well as its anti-neuroinflammatory effect, might be related to neuroprotective effect. Our findings add evidence to support the idea of the potential use of *Ocimum americanum* as a nutraceutical or functional food in the aging process.

## 1. Introduction

Functional foods, including berries and virgin olive oil, have raised attention for their health benefits in aging. Several phytochemicals, including those found in foods, seems to be able to counteract, at least partly, the aging-induced functional impairments, such as cognitive decline, biochemical, and molecular pathway modifications, and neurodegenerative events like chronic/persistent oxidative stress, neuroinflammation, blood–brain barrier, and mitochondrial dysfunctions [1].

There are a few studies on the potential benefits of Lamiaceae species during normal aging. Li and colleagues (2022) reported that *Scutellaria baicalensis* Georgi, using the aging model of the administration of D-galactose to young adult rats, has a potential anti-aging effect [2]. Some Lamiaceae species, such *Salvia* spp. L. and *Rosmarinus officinalis* L. (respectively, sage and rosemary) have been described as potential neuroprotective interventions in the aging process and neurodegenerative diseases, impacting relevant brain functions due to their antioxidant, cholinesterase inhibition, and anti-inflammatory properties [3,4]. Interestingly, sage and rosemary have been used to treat memory loss in European folk medicine. It is worth noting that a systematic review with clinical trials performed with healthy individuals or participants with cognitive impairment, including dementia conditions, concluded that *Salvia officinalis* L. and *S. lavandulaefolia* Vahl. may improve cognitive performance both in healthy and impaired subjects [5].

The neuroprotective properties of some members of the *Ocimum* genus (Lamiaceae) have also been studied in experimental cerebral ischemia models. Extracts of *Ocimum sanctum* L., a species widely used in the Ayurveda traditional medicine, commonly known as tulsi, protect against ischemia-reperfusion and hypoperfusion-induced cerebral damage by reducing the cell membrane dysfunction, specially that related to antilipoperoxidant activity [6]. Mataram and colleagues (2021) showed the potential neuroprotective effects of *O. sanctum* extract [7], and the treatment with *O. basilicum* L. was found to protect against ischemia-reperfusion and hypoperfusion-induced cerebral damage [8,9].

Recently, it was demonstrated that long-term supplementation of chow with sweet basil (*Ocimum basilicum*) leaves improved the memory performance of female aged mice in the novel object recognition test [10], and that its hydroethanolic extract administered for 8 weeks by oral gavage may improve learning and memory impairment in male aged rats [11].

The potential mode(s) of action(s) supporting neuroprotection induced by Lamiaceae species extracts and their compounds has been widely related to antioxidant and anti-inflammatory actions. In vitro antioxidant activity and neuroprotective effects of *O. sanctum* were observed on hydrogen peroxide-induced oxidative damage in SH-SY5Y human neurons, such as reducing the lipid peroxidation levels and reactive species generation [12]. It is important to highlight that hydrogen peroxide (H_2_O_2_) is a central reactive oxygen species, generated at low levels under normal metabolism; however, it is produced at higher levels under ischemia-reperfusion conditions. Indeed, Harman’s free radical theory of aging (1954) has been recognized as the most relevant in the context of brain aging; the role of accumulated oxidative markers over brain aging, including macromolecule damage, such as lipid peroxidation, protein, and DNA damage, has not been refuted so far [13]. In addition, it was demonstrated that *O. basilicum* has antioxidant properties in several in vitro systems [14]. Interestingly, Zengin and colleagues (2019) [15] showed that aqueous extract of *O. americanum* L. flowers and leaves had better in vitro antioxidant activity, followed by methanol extraction, determined by several methods, such as ABTS•+, DPPH•, CUPRAC, FRAP, metal chelating, and phosphomolybdenum. Authors suggested that the best radical scavenging potential of *O. americanum* aqueous extract was related to its richest profile of phenolic compounds [15].

Some studies pointed out that the normal aging process is associated with increased neuroinflammatory response, characterized by changes in the hippocampus pro-inflammatory cytokine levels, such as interleukin-1β (IL-1β) and tumor-necrosis factor alpha (TNF-α) [16,17]. Porcher and colleagues (2021) showed that hippocampi are more susceptible to increased cytokines produced by aging than the other brain regions [17]. *Ocimum* species modulation of neuroinflammation parameters has received more attention recently. *O. basilicum* extract reversed maternal separation stress-induced increases in TNF-α and IL-1β expression in the hippocampus [18], and *O. basilicum* hydroethanolic extract administered for 8 weeks (v.o.) reversed aging-induced effects on interleukin-6 (IL-6) levels in the hippocampus and cortex from aged rats [11].

There is sound evidence of cholinergic function impairments during normal aging, and of pronounced loss of cholinergic transmission in cognitive-involved neurodegenerative diseases, including Alzheimer’s disease (AD) [19]. Corroborating that, acetylcholinesterase (AChE) inhibition is a pharmacological strategy of early AD management. Several herbal medicines/medicinal plants/functional foods, and their bioactive compounds, have been described as AChE inhibitors. For example, *Uncaria tomentosa* (Willd. ex Roem. & Schult., Rubiaceae) DC., commonly known as “cat’s claw”, which is found in the Brazilian Amazon, improved memory performance and reduced the hippocampus AChE activity of middle-aged rats treated for 12 months [20]. Several *Salvia* species have demonstrated in vitro anticholinesterase activity, such as *Salvia lavandulaefolia, Salvia aristata*, and *Salvia leriifolia* [21,22,23], which has been related to their benefits on cognitive performance described above. *O. americanum* had the most potent in vitro AChE inhibition activity and, among the tested compounds, chlorogenic acid had remarkable action. In vitro findings also showed that methanol and ethyl acetate extracts of flowers or leaves of *O. americanum* L. were effective in inhibiting AChE, while the aqueous extract had insignificant activity [15]. Although there is considerable literature on in vitro AChE inhibition of Lamiaceae species and their compounds, few studies report in vivo anticholinesterase effects.

The present work studies *O. americanum* (“alfavaca”, “manjericão, “Americanum basil” or “hoary basil”), an aromatic annual herb, native to Africa, the Indian Subcontinent, China, and Southeast Asia, and naturalized in several countries [24]. Brazil has extensive influence of Indigenous peoples, Europeans, and Africans on the potential of natural resources, including the flora. In this context, there is evidence of the role of African slaves in the dissemination of *Ocimum* specimens over our country, where they were victims of forced labor [24].

Considering that several *Ocimum* species have shown preclinical neuroprotective actions, traditional uses are similar among the *Ocimum* species [24], and that *Ocimum americanum* has in vitro antioxidant and anticholinesterase activities [15], this work can open new insights bringing evidence as its potential use as a nutraceutical or functional food, considering its globally recognized culinary applications. Our hypothesis is that *O. americanum* is a neuroprotective species, especially in the aging process, by modulating anticholinesterase activity, oxidative stress, and/or inflammatory status using ex vivo and in vivo models.

The aim of the present study is to evaluate the neuroprotective effects of *Ocimum americanum* ethanol extract (OAEE) and its putative mode of action on hippocampus tissue from young adult and late middle-aged Wistar rats, focusing on oxidative stress, cholinesterase activity, and neuroinflammatory markers. Specifically, we investigated (1) the effects of OAEE incubation on hippocampal slices of young adult and late middle-aged rats exposed to oxidative stress (H_2_O_2_); (2) effects of an acute administration with OAEE on aversive memory performance and neurochemical parameters, as well as (3) the impact of a chronic administration with a diet supplementation with OAEE in hippocampus from young adult and late middle-aged male rats.

## 2. Materials and Methods

### 2.1. Plant Material and Preparation of the Ethanol Extract

*Ocimum americanum* (Lamiaceae) was collected in Rio Grande do Sul State (Brazil) and authenticated by Mara Rejane Ritter (Departamento de Botânica, Instituto de Biociências, UFRGS, RS, Brazil). Voucher specimens were deposited in the Herbario do Vale do Taquari (HVAT) Botany and Paleobotany Sector of UNIVATES Natural Science Museum (Lajeado, Rio Grande do Sul State, Brazil).

One hundred grams of fresh leaves of *O. americanum* was soaked in 1000 mL of ethanol (90%) for 7 days at room temperature. The ethanol extract, after evaporation, was kept in a vacuum desiccator until constant weight, resulting in the dried ethanol extract (OAEE). Total phenolic compound content was determined using the Folin–Ciocalteu reagent, following a slightly modified Singleton’s method using quercetin as standard [25]. The content of total phenolic compounds in our extract was 23.61 mg quercetin equivalents per gram of extract.

### 2.2. Animals

Male Wistar rats aged 3 and 16–18 months-old were used. The animals were maintained under standard conditions (12 h light/dark, 22 ± 2 °C) with food and water ad libitum. The Local Ethics Committee approved all handling and experimental conditions (GPPG-HCPA 09-638). Animals were provided by Centro de Reprodução e Experimentação de Animais de Laboratório (CREAL/UFRGS) and up to five (young adults) or three (aged rats) per cage housed (Plexiglass cages, dimensions: 40 × 33.3 × 17 cm) in standard housing and husbandry with wood shavings as bedding material of the SPF-grade animal laboratory. All manipulations and procedures, including gavage, were performed by trained researchers.

### 2.3. Ex Vivo Experiments

#### 2.3.1. Animals, Preparation, and Incubation of Hippocampal Slices

Male Wistar rats aged 3 and 16–18 months, respectively, n = 9 and 12, were decapitated, and the hippocampi were quickly dissected out, and transverse sections (400 µm) were prepared using a Stoelting™ McIlwain Tissue Chopper (Wood Dale, IL, USA).

Slices were then transferred immediately into 24-well plates, each well containing 0.3 mL of HEPES-saline buffer (containing in mM): 120 NaCl; 25 HEPES; 10 glucose; 2 KCl; 1 CaCl_2_; 1 MgSO_4_; and 1 KH_2_PO_4_; adjusted to pH 7.4. The medium was changed after 30 min by a fresh buffer and the slices were incubated with different concentrations of the OAEE (0, 0.1, and 1 μg/mL) for 60 min at 35 °C [26]. Each concentration was tested in triplicate and 12 slices per animal were used (n = 6–8).

The medium was then changed for a fresh buffer in the absence or presence of H_2_O_2_ (2 mM) for 60 min at 35 °C [26]. After incubation with H_2_O_2_, cellular viability (mitochondrial activity) and cell damage (membrane lysis) assays were performed. The incubation of hippocampal slices from both 3 and 16–18 month-old rats with H_2_O_2_ resulted in marked changes in cellular viability (MTT assay). H_2_O_2_ is widely used as an oxidative injury model in rat hippocampal slices [26]. For the ex vivo assays OAEE was firstly dissolved in 1% dimethyl sulfoxide (DMSO) and was further adjusted to final concentration; a maximum final concentration of DMSO in OAEE-treated samples was 0.001%, which was used as a control group (OAEE 0 μg/mL).

#### 2.3.2. Cellular Viability

Mitochondrial activity was evaluated by the colorimetric 3 (4,5-dimethylthiazol-2-yl)-2,5-diphenyl tetrazolium bromide (MTT) method. Hippocampal slices were incubated for 30 min at 35 °C in the presence of MTT (5 mg/mL). Active mitochondrial dehydrogenases of living cells cause cleavage and reduction in the soluble yellow MTT dye to the insoluble purple formazan, which was extracted in DMSO; the absorbance was measured at 560/630 nm [27].

#### 2.3.3. Cellular Damage

Cell damage was quantified by measuring released lactate dehydrogenase (LDH) into the medium. After 60 min of incubation with H_2_O_2_, LDH activity was determined using a commercial kit (Doles Reagents, Goiânia, Brazil). Each experiment was normalized by subtracting the background levels of control wells. Samples were quantified using a standard curve; the absorbance was measured at 490 nm [26].

### 2.4. In Vivo Acute Experiments

#### 2.4.1. Animals and Treatment

Adult male Wistar rats (90 days old and weighing approximately 250 g) were treated with saline, DMSO 20% (control group) or OAEE (100 or 300 mg/kg) by gavage (n = 6; total = 24 rats). OAEE doses (100 and 300 mg/kg) were selected based on the acute effects of hydroalcoholic extract of *Rosmarinus officinalis* (Lamiaceae) in a social recognition and an inhibitory avoidance task [28]. Animals received the treatments immediately after single training on inhibitory avoidance and were tested for short-term memory 90 min after training.

#### 2.4.2. Inhibitory Avoidance Task

The single-trial step-down inhibitory avoidance (IA) conditioning was employed as a model of fear-motivated memory. The IA behavioral training and retention test procedures were described in previous reports. The IA apparatus was a 50 cm × 25 cm × 25 cm acrylic box (Albarsch, Porto Alegre, Brazil) whose floor consisted of parallel caliber stainless steel bars (1 mm diameter) spaced 1 cm apart. A 7 cm wide, 2.5 cm high platform was placed on the floor of the box against the left wall. On the training trial, rats were placed on the platform and their latency to step down on the grid with all four paws was measured with an automatic device. Immediately after stepping down on the grid, rats received a 0.5 mA, 2.0 s foot shock and were removed from the apparatus. The test trials were procedurally identical to training, except that no foot shock was presented. Step-down latencies on the test trial (maximum 180 s) were used as a measure of IA retention. Behavioral observations were performed with the same procedural factors, such as illumination intensity, in soundproof rooms during the same period of the day. Rats were handled by the same researcher with well-trained skills to minimize stress, without prior handling.

#### 2.4.3. Tissue Preparation

Rats were decapitated 30 min after the test on inhibitory avoidance; hippocampi were quickly dissected out and instantaneously placed in liquid nitrogen and stored at −70 °C until biochemical assays.

For assays of cellular oxidative state, the hippocampi were homogenized in 10 volumes of ice-cold phosphate buffer (0.1 M, pH 7.4) containing ethylenediaminetetraacetic acid (EDTA, 2 mM) and phenylmethylsulfonyl fluoride (PMSF, 1 mM) in a Teflon-glass homogenizer. The homogenate was centrifuged at 960× *g* for 10 min and the supernatant was used for the assays [29]. Each batch was run with at least two samples of each group, including controls and standards (n = 6–8). To evaluate the acetylcholinesterase activity, the hippocampi were homogenized in 10 volumes of ice-cold phosphate buffer (0.5 M, pH 7.5) and centrifuged at 900× *g* for 10 min, and the resulting supernatant was used as enzyme source.

#### 2.4.4. Reactive Species Levels

2′-7′-dichlorofluorescein diacetate (DCFH-DA) was used as a probe to measure the reactive species content. An aliquot of the sample was incubated with DCFH-DA (100 μM) at 37 °C for 30 min. The formation of the oxidized fluorescent derivative (DCF) was monitored at excitation and emission wavelengths of 488 nm 525 nm, respectively. All procedures were performed in the dark and blanks containing DCFH-DA (no supernatant) were processed for measurement of autofluorescence [29].

#### 2.4.5. Thiobarbituric Acid Reactive Substances (TBARS)

Lipid peroxidation (LPO) was evaluated by a thiobarbituric acid reactive substances (TBARS) test [2]. Aliquots of samples were incubated with 10% trichloroacetic acid and 0.67% thiobarbituric acid. The mixture was heated (30 min) in a boiling water bath. Afterwards, n-butanol was added and the mixture was centrifuged. The organic phase was collected to measure fluorescence at excitation and emission wavelengths of 515 and 553 nm, respectively. 1,1,3,3-tetramethoxypropane, which was converted to malondialdehyde (MDA), was used as standard.

#### 2.4.6. Acetylcholinesterase Activity

Acetylcholinesterase (AChE) activity was determined by slight modifications of a colorimetric method described by Ellman and colleagues [30], using acetylthiocholine iodide (ASCh) as a substrate. The total volume of reaction mixtures was 300 µL (10 µL of supernatant, 30 µL of Ellman’s reagent [0.01 M 5-5′-dithio-bis(2-nitrobenzoic acid)], 30 µL of ASCh, 230 µL of buffer), and each sample of the enzyme source material was analyzed in triplicate. The blank reading was obtained for each reaction mixture after 10 min of incubation, before the addition of ASCh (75 mM). Thereafter, absorbance (412 nm) readings were taken for 4 min at 30 s intervals.

### 2.5. In Vivo Chronic Experiments

#### 2.5.1. Animals and EEOA Supplementation

Forty male Wistar rats aged 3 and 16–18 months were randomly divided into six groups and fed a diet with or without OAEE supplementation during 4 weeks.

The commercial non-purified chow diet from Nuvilab-CR1 (Curitiba, Brazil; caloric density 4.16 cal/g) that contained (g/100 g) total fat 11, protein 22, fiber 3 ash 6, carbohydrates 56, salts, and vitamins as recommended by the Association of Official Analytical Chemists, Washington, D.C. was used. The dried extract was mixed to ground commercial chow and reconstituted.

The animals were randomly assigned to one of three groups: the normal chow diet (0%, control diet) or experimental diet at low dose (chow supplemented with OAEE, at a final concentration of 0.1 g/kg, 0.01%) or 0.2% (chow supplemented with OAEE at a final concentration of 2 g/kg) [31]. The rats were fed ad libitum the standard rat chow or experimental diets (n = 6–8). The animals were observed daily for clinical signs of toxicity, and weekly for body weight changes, and mortality to clarify the toxicity profile of OAEE, as well as food consumption.

#### 2.5.2. Tissue Preparation

After 4 weeks of supplementation, the rats were decapitated and the hippocampi were quickly dissected out and instantaneously placed in liquid nitrogen and stored at −70 °C until biochemical assays. For the measurement of TNF-α and IL-1β content, the hippocampus was homogenized in phosphate-buffered saline (PBS, pH 7.4), containing 1 mM ethylene glycol tetraacetic acid (EGTA) and 1 mM PMSF. The homogenate was centrifuged at 1000× *g* for 5 min at 4 °C and the supernatant was used. For assessment of reactive species levels, thiobarbituric acid reactive substances and acetylcholinesterase activity, the homogenization of hippocampal samples and the procedures were performed as described in the section in vivo acute experiments.

#### 2.5.3. Measurement of Cytokines, TNF-α, and IL-1β Content

The Tumor Necrosis Factor-α (TNF-α) and interleukin-1β (IL-1β) levels in the hippocampus were determined using the Ready-To-Go Cytokine Elisa Kit (eBioscience, catalog number 88-7346 and 88-6010, respectively) according to the manufacturer’s protocol.

### 2.6. Protein Determination

Protein was measured by the Bradford method using Coomassie brilliant blue dye and bovine serum albumin as standard [32].

### 2.7. Statistical Analysis

The sample size was calculated using G*Power software (version 3.1) with 80% power and 0.05 significance level (one-tailed), based on an expected difference of 40% on MTT levels (ex vivo assay), TNF-α contents (acute administration and supplementation effects). Based on these parameters, the minimum sample size was set at n = 5/group; however, it was adjusted between n = 5–8 per group, anticipating some sample loss (sample storage, assay preparation, operational failure or mistakes). In addition, for the statistical analysis, a few data points were excluded such as events of technical errors (negative data), clearly resulting from improper sample processing.

The Kolmogorov–Smirnov test and Levene’s test were, respectively, used to evaluate the data distribution pattern and variance homogeneity in the data. The Kolmogorov–Smirnov test indicated that the inhibitory avoidance task and acetylcholinesterase activity data had nonparametric distributions, where Kruskal–Wallis was employed. Results are expressed as percentage of control and are represented as mean (±SEM). The ex vivo neuroprotective effects were evaluated by Two-way ANOVA followed by Tukey’s test with H_2_O_2_ exposure and treatments as factors. The effect of acute treatment was evaluated by One-way ANOVA and a post hoc Tukey test. The chronic supplementation effects were evaluated by Two-way ANOVA followed by Tukey’s test with age and supplementation as factors. We estimate the effect size using Cohen’s d. Statistical Package for the Social Sciences (SPSS 15) software was used. In all tests, *p* < 0.05 was considered to indicate statistical significance.

## 3. Results

### 3.1. Ex Vivo Neuroprotective Effect of Ocimum Americanum Extract in Young Adult and Late Middle-Aged Rats

**Figure 1:**
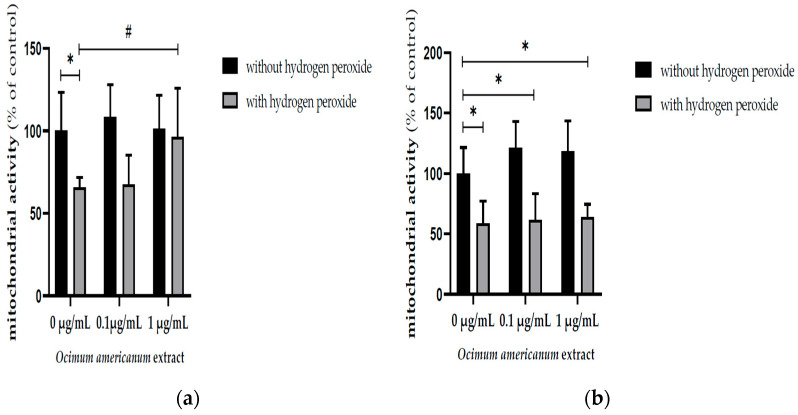
Effect of incubation with *Ocimum americanum* extract (0.1 and 1 µg/mL) on cellular viability in hippocampal slices submitted to H_2_O_2_, evaluated by MTT assay. (**a**) Young adult rats (3 months, control without H_2_O_2_ n = 7; 0.1 µg/mL n = 6; 1 µg/mL n = 5; control with H_2_O_2_ n= 6; 0.1 µg/mL n = 7; 1 µg/mL n = 6); (**b**) late middle-aged rats (16–18 months, control without H_2_O_2_ n= 8; 0.1 µg/mL n = 7; 1 µg/mL n = 7; control with H_2_O_2_ n = 8; 0.1 µg/mL n = 7; 1 µg/mL n = 7). Results were expressed as percentage of control group (0 µg/mL without H_2_O_2_) and analyzed by Two-way ANOVA followed by the Tukey test. * and # *p* < 0.05; * = values significantly different from control group; # = values significantly different from 0 µg/mL with H_2_O_2_.

The H_2_O_2_ incubation significantly reduced the mitochondrial activity in hippocampal slices from young adult and late middle-aged rats (Figure 1a, F_(1,36)_ = 22.164, *p* < 0.0001; Figure 1b, F_(1,44)_ = 93.416, *p* < 0.0001, respectively) evaluated by MTT assay. The incubation with 1 µg/mL OAEE impacted this parameter in hippocampal slices from young adult rats (*p* = 0.05; Cohen’s *d* = 1.634, large effect size), without any significant effect in aged rats. In accordance, there was a significant interaction between two factors, H_2_O_2_ and OAEE (F_(2,36)_ = 5.530, *p* = 0.042).

**Figure 2:**
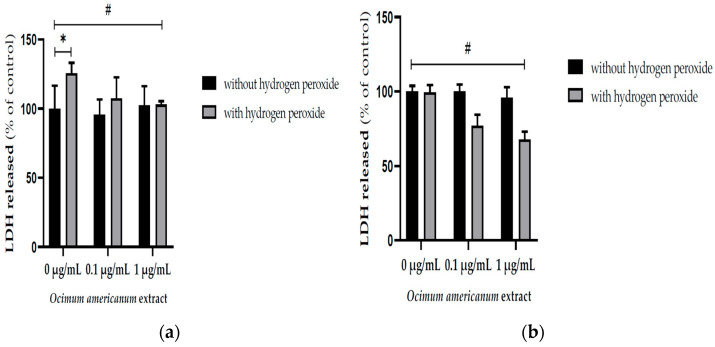
Effect of incubation with *Ocimum americanum* extract (0.1 and 1 µg/mL) on cellular damage in hippocampal slices submitted to H_2_O_2_, evaluated by released LDH: (**a**) young adult rats (3 months, control without H_2_O_2_ n = 6; 0.1 µg/mL n = 5; 1 µg/mL n = 5; control with H_2_O_2_ n = 6; 0.1 µg/mL n = 5; 1 µg/mL n = 5); (**b**) late middle-aged rats (16–18 months, n = control without H_2_O_2_ n = 8; 0.1 µg/mL n = 7; 1 µg/mL n = 6; control with H_2_O_2_ n = 6; 0.1 µg/mL n = 5; 1 µg/mL n = 6). Results are expressed as percentage of control group (0 µg/mL without H_2_O_2_) and analyzed by Two-way ANOVA followed by the Tukey test. * and # *p* < 0.05; * = values significantly different from the control group; # = values significantly different from 0 µg/mL with H_2_O_2_.

The exposure to H_2_O_2_ enhanced the LDH released into the incubation medium by hippocampal slices from young adult rats (Figure 2a, F_(1,33)_ = 14.532, *p* = 0.001). Interestingly, the incubation with 1 μg/mL OAEE reversed H_2_O_2_ -induced increases in LDH released by slices from young adult (F_(2,33)_ = 4.956, *p* = 0.014) and aged rats (F_(2,41)_ = 5.315, *p* = 0.009). Large effect sizes were observed in both young adult and aged samples; respectively, Cohen’s *d* were 14.14 and 6.4.

### 3.2. Effect of Acute Treatment with Ocimum Americanum Extract in Young Adult Rats

**Figure 3:**
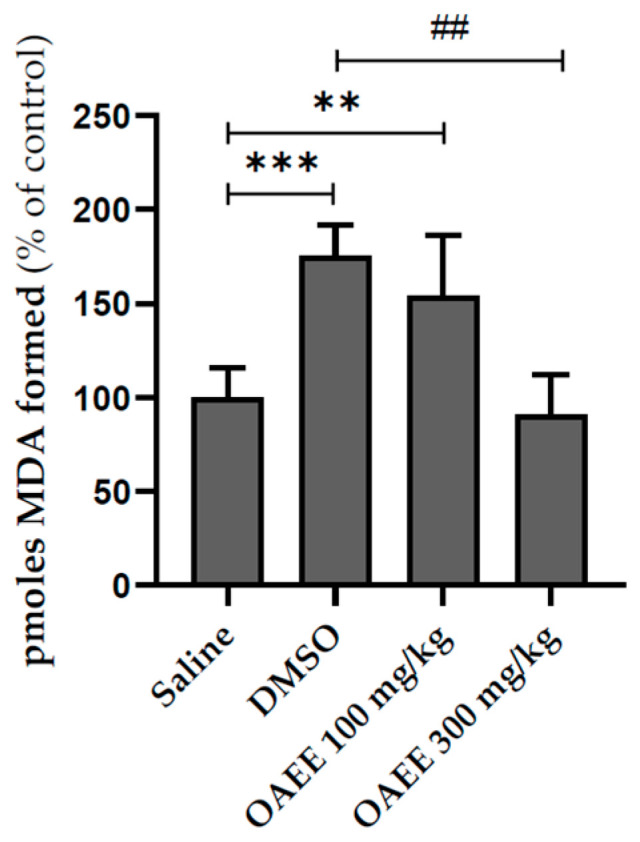
Effect of acute treatment with *Ocimum americanum* extract on LPO levels in hippocampus from young adult rats (3 months, saline n = 6; DMSO n = 5; 100 mg n = 5; 300 mg n = 5). Results are expressed as percentage of control (saline group) and analyzed by One-way ANOVA followed by the Tukey test. ** or ## *p* < 0.01; *** *p* < 0.001; * = values significantly different from the saline group; ## = values significantly different from the DMSO group.

Latencies to step down from the platform during training did not differ among groups. The immediately post-training acute treatment with OAEE did not alter the short-term aversive memory evaluated in the inhibitory avoidance task. The acute treatment with OAEE did not modify the species reactive levels, evaluated by formation of the DCF, and the AChE activity in the hippocampus from young adult rats. The administration (v.o.) of DMSO significantly increased the LPO levels when compared to the saline group, and 300 mg/kg OAEE was able to reverse this effect to control levels (Figure 3, F_(3,20)_ = 17.78; *p* < 0.0001). Cohen’s *d* indicates a large effect size (*d* = 4.5).

### 3.3. Effect of Ocimum Americanum Extract Supplementation in Young Adult and Aged Rats

**Figure 4:**
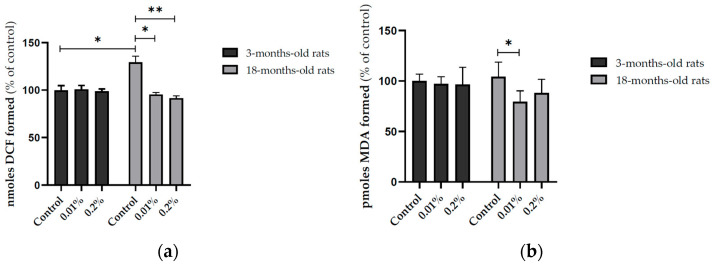
Effect of diet supplementation with *Ocimum americanum* extract on cellular oxidative state in the hippocampus from young adult (3-month-old rats) and late middle-aged rats (16–18-month-old). (**a**) DCF levels (YA control n = 7; 0.01% n = 6; 0.2% n = 8; aged control n = 6; 0.01% n = 7; 0.2% n = 6); (**b**) LPO levels (YA control n = 5; 0.01% n = 5; 0.2% n = 6; aged control n = 5; 0.01% n = 6; 0.2% n = 5). Results are expressed as percentage of control (young control group) and analyzed by the Two-way ANOVA followed by the Tukey test; * *p* < 0.05, ** *p* < 0.01.

The supplementation with OAEE (0.01 and 0.2%) during 4 weeks induced a large effect on reactive species content in the hippocampus of late middle-aged rats, compared to those obtained from the respective control group (Cohen’s *d* = 6.156 and 9.689; Figure 4a, *p* < 0.0001). An age-dependent effect on reactive species levels was found (Figure 4a, F_(1,39)_ = 4.311, *p* = 0.046), because the OAEE did not modify the reactive species content in the hippocampus from young adult rats. In accordance, there was a significant interaction between two factors, age and OAEE supplementation (F_(2,39)_ = 18.669, *p* < 0.0001). The supplementation with 0.01% OAEE reduced the TBARS levels in aged rats (Figure 4b, *p* = 0.032, Cohen’s *d* = 2.055, a large effect size). No significant difference induced by OAEE supplementation was observed in young adult rats, compared with the control group. Furthermore, two-way ANOVA showed the effect of OAEE supplementation (F_(2,31)_ = 3.354, *p* = 0.051).

**Figure 5:**
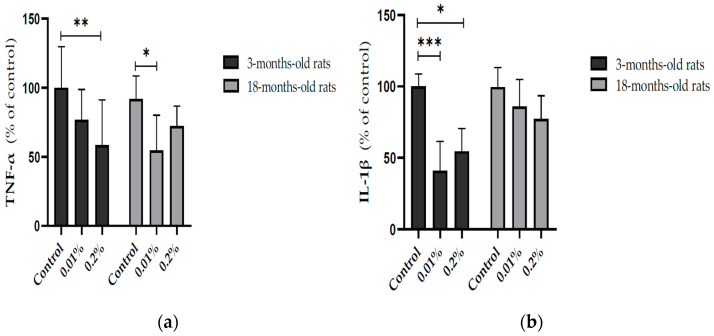
Effect of diet supplementation with *Ocimum americanum* extract on neuroinflammatory parameters in the hippocampus from young adult (3-month-old rats) and late middle-aged rats (16–18-month-old). (**a**) TNF-α content (YA control n = 6; 0.01% n = 6; 0.2% n = 5; aged control n = 4; 0.01% n = 5; 0.2% n = 6); (**b**) IL-1β content (YA control n = 5; 0.01% n = 5; 0.2% n = 5; aged control n = 6; 0.01% n = 6; 0.2% n = 5). Results are expressed as percentage of control (young control group) and analyzed by the Two-way ANOVA followed by the Tukey test; * *p* < 0.05; ** *p* < 0.01; *** *p* < 0.001.

In addition, the modulation of OAEE supplementation on neuroinflammatory processes in the hippocampus from young adult and aged rats was evaluated. The OAEE reduced the content of TNF-α in the hippocampus from young adult and late middle-aged rats, since two-way ANOVA showed the effect of OAEE supplementation (Cohen’s *d* = 1.354 and 1.811, respectively; F_(2,27)_ = 8.421, *p* = 0.002, Figure 5a). The OAEE (0.01% and 0.2%) decreased the content of IL-1β in the hippocampus; however, there was an interaction between two factors, age and OAEE supplementation (F_(2,31)_ = 5.296, *p* = 0.012, Figure 5b) because major changes were induced in young adult (Cohen’s d were 3.939 and 3.637, while 0.2% aged had 1.509).

## 4. Discussion

The presented findings support the hypothesis that the extract of *O. americanum* has neuroprotective action, especially in the aging process. Although the current state of knowledge concerning plant-based compounds as senolytic agents has been recognized, there was a lack of reports on *Ocimum americanum* in rat models. To our knowledge, this is the first study evaluating the neuroprotective potential of *O. americanum* in the context of aging. While similar effects have been reported for *Ocimum sanctum* and *Salvia officinalis*, the present findings extend this knowledge to a less studied species, suggesting a broader neurotherapeutic potential within the Lamiaceae family. Mechanistically, *Ocimum americanum*-mediated neuroprotection can be attributed to its antioxidant and anti-neuroinflammatory actions.

Chronic supplementation with OAEE for 4 weeks was able to reduce the age-induced increase in reactive species levels. In accordance with previous results, reactive species content was significantly increased in the aged hippocampus [29]. The essential oil and aqueous and methanol extracts of *O. americanum* leaves, determined by several methods, had in vitro free radical scavenging activities [15,33]; the major compounds of essential oil are camphor, limonene, longifolene, and caryophyllene [33]. However, Zengin and colleagues suggested that the best radical scavenging potential of *O. americanum* aqueous extract was related to its richest profile of phenolic compounds, phenolic acids, flavonoids and tannins, among them, gallic acid, caftaric acid, chlorogenic acid, caffeic acid, rosmarinic acid, vitexin, isovitexin, rutin, luteolin, and apigenin [15].

It is relevant to comment that the accumulated macromolecules damage, such as lipid peroxidation, is pronounced in the aging process [13], including in the aged hippocampus [29]. There is consistent evidence showing the role of Lamiaceae species on lipid peroxidation, specially TBARS levels. Here, an acute administration of OAEE in young adult rats was able to reverse increases in LPO induced by DMSO (TBARS levels), DMSO can exhibit a pro-oxidant action [34]. Accordingly, chronic administration of the *O. americanum* extract was able to decrease the hippocampal TBARS levels of 16–18-month-old rats. These findings are in agreement with those that describe the effect of other *Ocimum* species preventing the LPO induced by ischemia/reperfusion and noise exposure [6,8,35]. Treatment with *Ocimum basilicum* hydroethanolic extract (150 mg/kg, v.o.) for 8 weeks prevented learning and memory impairment and decreased hippocampal and cortical TBARS levels in male aged rats [13]. Therefore, it is possible to suggest that the effect of OAEE on H_2_O_2_ –induced cell damage can be due, at least in part, with its antilipoperoxidant activity.

*O. americanum* extract was able to protect cell membranes, since a reduction in released LDH in hippocampal slices from both young adult and late middle-aged rats was observed, while mitochondrial activity (MTT assay) was improved only in young adult rats, without any significant effect in late middle-aged ones. Accordingly, the neuroprotective action of some species of the Lamiaceae family against the oxidative damage induced by H_2_O_2_ in different cell types has been described [12,36,37]. Asadi and colleagues [36] demonstrated that the pre-treatment with *Salvia* species from Iran significantly protected neuron-like PC12 cells against H_2_O_2_ -induced toxicity. *Rosmarinus officinalis* was effective in attenuating the disruption of mitochondrial membrane and cell death induced by H_2_O_2_ in SH-SY5Y cells [36]. It is relevant to consider that, even in the aging process, OAEE had beneficial effects on cellular membranes. Our ex vivo data may be related to other findings here described, since both acute and chronic treatments reduced LPO levels.

In addition to free radical scavenging effects, several mechanisms may have been involved with the neuroprotective effects of *Ocimum* species. The cortical lipidomic profiles of mice submitted to a middle cerebral artery occlusion, modeling photothrombotic stroke, were altered by oral administration of *Ocimum sanctum* ethanol extract for seven days [38]. The extract- and ibuprofen-treated mice had 13 common upregulated lipid species, such as dimethyl-phosphatidylethanolamine (18:1/14:0), phosphatidyl-ethanolamine (16:0/22:4), sphingomyelin (d44:7), phosphatidylcholine (17:0), phosphatidyl-ethanolamine (43:10), monogalactosyl-monoacylglycerol (14:2), di-galactosyl diacylglycerol (42:9), phosphatidylinositol (28:5), phosphatidylserine (8:0e/21:6), lysophosphatidylcholine (16:1), and phosphatidyl-ethanolamine (39:6), while both groups showed downregulated lipid species, phosphatidyl-ethanolamine (35:0p), and di-galactosyl diacylglycerol (42:14) [38]. This change in lipidomic profiles could be involved with anti-inflammatory, antilipoperoxidant effects, and cell membrane integrity.

Another novel finding from our study is the interaction between *O. americanum* and neuroinflammatory parameters. The chronic supplementation with OAEE modulated pro-inflammatory cytokine levels in the hippocampus from 3- and 16–18-month-old rats. Namely, OAEE was able to reduce the TNF-α content in 3- and 16–18-month-old rats, as well as the IL-1β levels in hippocampus from 3-month-old rats, with a modest impact in late middle-aged animals. These results are relevant since it was described that the inflammatory cytokine profile may be involved, at least in part, in the aging-related impairments [16].

AChE inhibition of Lamiaceae species has been related to central effects, since its higher activity may imply increased levels of acetylcholine and, consequently, improvements of cholinergic neurotransmission that is relevant in the management of Alzheimer’s disease. Farag and colleagues (2016) [39] compared bioactive metabolites, using metabolomic techniques, and an in vitro anticholinesterase action of *O. americanum*, *O. africanum* Lour., *O. basilicum*, and *O. minimum* L. methanol extracts. Farag and colleagues (2016) showed that the methanol extract of *O. americanum* induced more potent in vitro AChE inhibition than those of *O. africanum* or *O. basilicum* [39]. In addition, methanol or ethyl acetate extracts of flowers or leaves of *O. americanum* were effective in inhibiting AChE, while aqueous extract had insignificant activity [15]. Hydroxycinnamates putatively mediate the in vitro anticholinesterase effect of tested *Ocimum* extracts; evaluated compounds, caftaric, chlorogenic, and rosmarinic acids also showed this property [39]. This work reported the qualitative abundance of hydroxycinnamates, monocaffeoyltartaric acid (caftaric acid), rosmarinic acid-O-glucoside, feruloyltartaric acid, feruloyl glucoside, rosmarinic acid derivative, dicaffeoyltartaric acid (cichoric acid), salvianolic acid B, rashomonic acid C/D, rosmarinic acid, and rosmarinic acid dimer [39]. Interestingly, fatty acids were more abundant in *O. basilicum* and *O. africanum* methanol extracts [39], which could improve the absorption of fat-soluble bioactive compounds. The low availability of AChE inhibitors of *O. americanum* into the CNS might explain our findings, because both acute treatment and chronic supplementation with *O. americanum* extract did not alter adult and aged hippocampal AChE activity.

There are only a few studies reporting on in vivo anticholinesterase effects of Lamiaceae species. Interestingly, ethanol and aqueous extracts of *Ocimum sanctum* improved memory performance evaluated by a passive avoidance task and reduced the AChE activity in different brain areas, specifically in the cortex, cerebellum, medulla oblongata, and midbrain region, of rats submitted to maximal electroshock, atropine or cyclosporine [40]. However, this work did not evaluate the impact on the hippocampus, consequently we cannot exclude that *O. americanum* could inhibit cortical AChE. On the other hand, Tadros and colleagues (2014) compared the in vitro and in vivo effects on anticholinesterase activity of essential oils obtained from *Ocimum basilicum* and *O. africanum* [41]. Both volatile oils and their major constituents, linalool, 1,8-cineol, eugenol, and camphor, showed in vitro anticholinesterase activities. Indeed, the treatment with *O. basilicum* and *O. africanum* inhibited the brain AChE activity and improved the memory performance in scopolamine-treated mice [41]. However, the highest in vitro effect was found with 1,8-cineol, while the administration of mice with this compound (30 mg/kg) did not impact brain AChE activity [41], in accordance with the hypothesis that even with in vitro evidence, some compounds of *O. americanum* can have low central availability.

However, considering that significant cholinergic neuron loss occurs predominantly in pathological aging, such as in Alzheimer’s disease, it is possible that the lack of central inhibition effects may avoid some side effects. In this way, we could infer its effects in a scopolamine-induced rodent model. Although cholinergic loss seems not to be an aging mark, aging causes gradual cholinergic dysfunction characterized by several neurotrophic impairments, dendritic and axonal degeneration, as well synaptic loss [42]. Chiang and colleagues (2021) demonstrated that a sage extract (*Salvia officinalis*) was able to induce Brain-derived neurotrophic factor (BDNF) expression, a signaling protein related to neuroprotection and memory performance [43]. This work opened new perspectives, including the involvement of neurotrophins as a mode of action [43].

It is remarkable to cite that our extract was not able to affect aversive memory performance in an acute administration protocol using young adult animals. This memory paradigm is related to fear conditioning training, since learning context is an aversive stimulus (footshocks). This is the initial processing of information, which makes it unworkable to use any analgesia protocol in the inhibitory avoidance task. In addition to non-significant effects on aversive task, the lack of behavioral or cognitive function evaluation in the diet supplementation study was based on the fact that exposure to memory paradigm per se may modulate neurochemical parameters [44,45]; in order to avoid this factor, the animals were not exposed to aversive learning context. However, we cannot exclude its effects on different memory paradigms not related to learning context with aversive stimulus (footshocks) in the aging process. Accordingly, supplementation or oral gavage with *Ocimum* spp. have improved memory performance in the novel object recognition test or Morris water maze in aged rodents, assessing, respectively, recognition and spatial learning and memory [10,11].

Although some culture cell models could be used to evaluate antioxidant and anti-inflammatory effects instead of hippocampal slices, we selected rat forebrain slices because of some advantages such as preserved cells, neurons, and glia cells with their connections [46]. In addition, brain functions and neuroprotection studies using animals cannot easily be replaced by better brain models modeling this complex and dynamic network system. Rodents have been widely used as a model of “normal” aging, since age-related declines in types of memories and brain function in both humans and animal models have been described, indicating good construct validity and the translational potential of the findings [47].

The ethanol extract of *Ocimum americanum* seems to bear neuroprotective compounds. Although, at this point, neither the active compound(s), by using a crude extract, nor the exact mechanism(s) by which *O. americanum* extract exerts its activities are completely known, it is fair to assume that the activities here described for OAEE can be related at least in part to the presence of phenolic compounds, which are widespread in the Lamiaceae family. Then, the lack of phytochemical characterization is a central limitation in this study.

## 5. Conclusions

In conclusion, the antioxidant properties—particularly the inhibition of lipid peroxidation—and the anti-neuroinflammatory effects may contribute to the reported neuroprotective activity of *Ocimum americanum* extract. This study provides the first in vivo evidence to support the potential use of *O. americanum* as a nutraceutical or functional food to mitigate age-related neurodegenerative changes. However, the study is limited by the absence of behavioral or cognitive assessments, and the absence of a phytochemical profile, such as chromatograms; consequently the active compounds responsible for these effects remain to be identified. Further studies should aim to isolate these bioactive constituents, elucidate their molecular mechanisms of action underlying their neuroprotective effects, and evaluate their efficacy in behavioral and clinical models of age-related cognitive decline.

## Data Availability

The data that support the findings of this study are available from the corresponding author upon reasonable request.

## Abbreviations

The following abbreviations are used in this manuscript:

OAEE	*Ocimum americanum* ethanol extract
MTT	3(4,5-dimethylthiazol-2-yl)-2,5-diphenyl tetrazolium bromide
LDH	lactate dehydrogenase
EDTA	ethylenediaminetetraacetic acid
PMSF	phenylmethylsulfonyl fluoride
DCFH-DA	2′-7′-dichlorofluorescein diacetate
LPO	lipid peroxidation
TBARS	thiobarbituric acid reactive substances
MDA	malondialdehyde
AChE	acetylcholinesterase
ASCh	acetylthiocholine iodide
PBS	phosphate-buffered saline
EGTA	ethylene glycol tetraacetic acid
IL-1β	interleukin-1β
TNF-α	Tumor Necrosis Factor-α
